# Network meta-analysis of Chinese herbal injections combined with the chemotherapy for the treatment of pancreatic cancer

**DOI:** 10.1097/MD.0000000000007005

**Published:** 2017-05-26

**Authors:** Dan Zhang, Jiarui Wu, Shi Liu, Xiaomeng Zhang, Bing Zhang

**Affiliations:** Department of Clinical Chinese Pharmacy, School of Chinese Materia Medica, Beijing University of Chinese Medicine, Beijing, China.

**Keywords:** Chinese herbal injections, network meta-analysis, pancreatic cancer, systematic review

## Abstract

**Background:**

This study sought to use a network meta-analysis to assess the effectiveness and safety of Chinese herbal injections (CHIs) combined with the chemotherapy for the treatment of pancreatic cancer.

**Methods:**

Randomized controlled trials (RCTs) regarding CHIs to treat pancreatic cancer were searched in PubMed, the Cochrane library, Embase, the China National Knowledge Infrastructure Database (CNKI), the Wan-Fang Database, the Chinese Scientific Journals Full-text Database (VIP), and the Chinese Biomedical Literature Database (SinoMed) up to November 2016. The quality assessment was conducted by the Cochrane risk of bias tool and network meta-analysis was performed to compare the effectiveness and safety of different CHIs combined with the chemotherapy. Data were analyzed using STATA 12.0 and Win-BUGS 1.4 software.

**Results:**

A total of 278 records were searched, and 22 eligible RCTs involving 1329 patients and 9 CHIs were included. The results of the network meta-analysis demonstrated that compared with the chemotherapy alone, Compound Kushen, Kangai or Kanglaite injection combined with chemotherapy yielded significantly higher probability of improving performance status. Aidi injection combined with chemotherapy was more effective in relieving leucopenia than using chemotherapy single. And these between-group differences were statistically significant. However, CHIs combined with chemotherapy could not achieve a better effect in the total clinical effect, nausea and vomiting. As for the cluster analysis for the adverse reactions (ADRs), the chemotherapy alone and Huachansu injection combined with the chemotherapy were inferior to relieve ADRs than the other CHIs plus chemotherapy for patients with pancreatic cancer.

**Conclusions:**

The current evidence showed that using CHIs on the basis of the chemotherapy could be beneficial for patients with pancreatic cancer in improving performance status and reducing the ADRs.

## Abstract 2

Supplemental Digital Content is available in the text

## 1 Introduction

Pancreatic cancer is one of the common malignancies in the digestive system.^[1]^ According to the World Health Organization (WHO) statistics, the global morbidity and mortality of pancreatic cancer respectively account for the first 13, 7 in 2008.^[2]^ And in China, the morbidity and mortality of pancreatic cancer ranks 7 and 6 of all malignant tumors.^[3]^ About 60% patients with pancreatic cancer may occur distant metastasis of tumor when they are diagnosed, and the median survival time is only 6 to 15 months, 5-year survival rate is close to 5%.^[4,5]^ The results of the present studies clarify that the incidence of pancreatic cancer is related to smoking, alcohol, diabetes, dietary habits, and other factors.^[6–11]^ In traditional Chinese medicine (TCM) theory, pancreatic cancer belongs to “lump in the abdomen causing distension and pain” or “mass located in the upper or lower abdomen,” the lump in the abdomen, jaundice, and pain appears as its main manifestation in clinic.^[12,13]^ TCM serve as an important part of the comprehensive treatment for pancreatic cancer, it not only can enhance immune function and anti-tumor ability, reduce the toxicity of radiotherapy and chemotherapy; but also can improve the clinical symptoms and performance status of patients with pancreatic cancer, furthermore, it is possible to prolong the survival time.^[3]^ Apart from the using TCM decoction of treatment based on syndrome differentiation, CHIs have the advantages of rapid absorption and high-bioavailability, and widely used in clinics. Especially, CHIs play an important role of anti-cancer in the treatment of tumor diseases by reducing toxicity, enhancing efficiency when it combined with radiotherapy and chemotherapy.^[14,15]^ Such as Kanglaite, Kangai, and compound Kushen injection can improve performance status, alleviating symptoms, control the development and metastasis of tumors.^[16–19]^

Currently, the majority of clinical trials are 2-arm trials which focus on the comparisons between CHIs plus chemotherapy and chemotherapy; however, the head-to-head comparisons of different CHIs about their effectiveness and safety are relatively lack.^[20]^ Network meta-analysis allows for the simultaneous evaluation of multiple interventions, and provides valuable information for clinical decision-making through indirect comparisons in the absence of direct comparisons.^[21,22]^ In addition, network meta-analysis can sort the different interventions based on their therapeutic effect and the probability of optimal interventions.^[23]^ CHIs plus the chemotherapy has been in the clinical application of patients with pancreatic cancer, while there is a lack of high-level evidence-based medical researches about it recently. Given above, this study sought to use a network meta-analysis to assess the effectiveness and safety of CHIs combined with the chemotherapy for pancreatic cancer.

## 2 Methods

This study, including its inclusion and exclusion criteria, literature search, data extraction, quality assessment, and statistical analysis components, was conducted in accordance with Cochrane criteria and PRISMA guidelines.

### 2.1 Inclusion and exclusion criteria

Only RCTs meeting the following criteria were included in this network meta-analysis: (1) Study type: RCTs regarding CHIs were combined with the chemotherapy for the treatment of pancreatic cancer, with irrespective of blinding methods the publishing language. (2) Study objects: the included participants met the pathological or cytological diagnostic criteria of pancreatic cancer, and no gender, race, or nationality limitations were imposed. And patients were without contraindications to chemotherapy and obvious abnormalities in their electrocardiograms and liver and kidney functions. (3) Interventions: The CHIs group was treated by CHIs plus the chemotherapy, while the chemotherapy group solely used the chemotherapy. The chemotherapeutic drugs included gemcitabine (GEM), docetaxel (DOC), tegafur (S1), cisplatin (DDP), oxaliplatin (L-OHP), 5-fluorouracil (5-Fu), leucovorin (LV). (4) Outcomes: The primary outcomes of the research were the clinical effectiveness rate and the performance status. The criterion of the rapeutical effect met the WHO for solid tumors released in 1979.^[24]^ The clinical effectiveness rate was calculated by the following formula: the clinical effectiveness rate = [number of complete response (CR) patients + partial response (PR)]/ total number of patients × 100%. The clinical effectiveness rate was defined as a complete response (CR) or a partial response (PR). Performance status was assessed by the Karnofsky performance score (KPS), which was calculated as follows: KPSs that increased by ≥10 points after treatment were considered to improve performance status; KPSs that decreased by ≥10 points after treatment were considered to lower performance status, and KPSs that increased or decreased by <10 points were considered stable. The secondary outcomes were the ADRs involving leucopenia, nausea and vomiting. And the criterion of the ADRs met the WHO for common toxicity criteria of chemotherapy drugs released in 1981.^[25]^ The incidence of ADRs was calculated by the following formula: the incidence of ADRs = (number of patients occurred ADRs)/ total number of patients × 100%.

RCTs meeting the following criteria were excluded in this network meta-analysis: (1) study type: as for the repeatedly published studies, only retaining the latest or more comprehensive ones. (2) Study objects: the patients suffered from other primary tumors, obstructive jaundice, or severe infections. (3) Interventions: the joints interventions were not chemotherapy (radiotherapy, hyperthermia, interventional therapy, etc.). CHIs’ route of administration was not intravenous infusion. The information of chemotherapeutic drugs, dose and duration of treatment was incomplete or incorrect. (4) Outcomes: the rapeutical effect or ADRs of RCTs was not in accordance with the criterion of WHO, and RCTs did not report the clinical effectiveness rate, the improvement of performance status and ADRs.

### 2.2 Literature search

RCTs involving regarding CHIs to treat pancreatic cancer were retrieved by searching the following databases from January 1979 to November 2016: PubMed, the Cochrane library, Embase, CNKI, VIP, CBM and the Wan-Fang Database. In English databases, the searching words in the pancreatic cancer category were “Pancreatic Neoplasms, Insulinoma, Gastrinoma, Glucagonoma, Somatostatinoma, Vipoma, Pancreatic Neoplasm, Pancreas Neoplasm∗, Pancreas Cancer∗, Pancreatic Cancer∗.” The specific Chinese and English search terms for each CHIs and specific retrieval strategies were shown in Attachment 1.

### 2.3 Data extraction and quality assessment

Two researchers (DZ and JW) independently read the titles and abstracts of the identified RCTs, excluding not relevant ones, reviews, and pharmacological experiments. Full texts of the possibly relevant RCTs were checked to further determine whether they met the inclusion criteria. The reference sections of the retrieved articles and meeting abstracts were also screened. Case reports, animal experiments, editorials, letters, and review articles were excluded. In the case that 1 publication overlapped with another publication of the same trial, only the article with more detail or the most recent article was included. The two researchers (DZ and JW) conducted their quality evaluations independently. If there was disagreement occurred, discussion or further inquiry to a third researcher (SL) was chosen as a way to decide. The main components of the extracted data were as follows: (1) general information: title, authors’ names, publication date, and literature sources; (2) patient information: the number of patients, patient ages, patient genders, patients’ KPSs before treatment, tumor types, and tumor stages; (3) intervention: the names, dosages, and treatment cycles of CHIs; (4) outcomes: the measured data about clinical effectiveness rate, performance status and ADRs.

The quality assessment was conducted by the Cochrane risk of bias tool, which included random sequence generation, allocation concealment, blinding of participants and personnel, blinding of outcome assessment, incomplete outcome data, selective outcome reporting, and other sources of bias.^[26]^ Each RCT was rated “high,” “unclear,” or “low.” “High” meant incorrect random methods, no allocation concealment or no blinding. “Unclear” meant no description in the text with which to assess bias. “Low” meant that a detailed description of correct random methods, the appropriate blinding without being violated through implementation.

### 2.4 Statistical analysis

First, the chi-squared test was used to evaluate heterogeneity among studies, and *I*^2^ was used to show the magnitude of this heterogeneity. Results of *P* ≥ .1 and *I*^2^ ≤ 50% suggested a lack of significant heterogeneity; in such cases, the Mantel-Haenszel fixed-effects model was used for meta-analysis. For cases with *P* < .1 and *I*^2^ > 50%, we explored sources of heterogeneity via subgroup analysis and meta-regression. When no clinical heterogeneity was indicated, the Mantel-Haenszel random-effects model was used to perform the meta-analysis.^[27]^ Moreover, the evaluation of the inconsistency between direct and indirect comparisons was unnecessary because a loop connecting the 3 arms did not exist in our study. Second, all calculations and graphs were made using STATA 12.0 software (Stata Corporation, College Station, TX), and Markov Chain Monte Carlo (MCMC) methods were performed using Win-BUGS 1.4.3 software (MRC Biostatistics Unit, Cambridge, UK). The network graph presented indirect comparative relationship between different interventions by STATA software.^[28]^ The data were analyzed by the Win-BUGS software, the posterior probability was deduced from the prior probabilities, and conducted the Bayesian inference under the assumption that the MCMC had reached a stable convergence state.^[29]^ Setting the number of iterations was 200,000, the first 10,000 for the annealing algorithm in order to eliminate the influence of initial value when running the Win-BUGS program. Third, dichotomous data was presented as the odds ratios (OR) and 95% confidence intervals (CI), and OR value used the median. Finally, we applied surface under the cumulative ranking probabilities (SUCRA) values to rank the examined treatments, with SUCRA values of 100% and 0% assigned to the best and worst treatments, respectively.^[30,31]^ Besides, a comparison-adjusted funnel plot was used to test for the publication bias. Furthermore, we used clustering methods and 2-dimensional plots to produce clusters of treatments to account for the incidence of ADRs.

## 3 Results

### 3.1 Literature search and the characteristics of included RCTs

**Figure 1:**
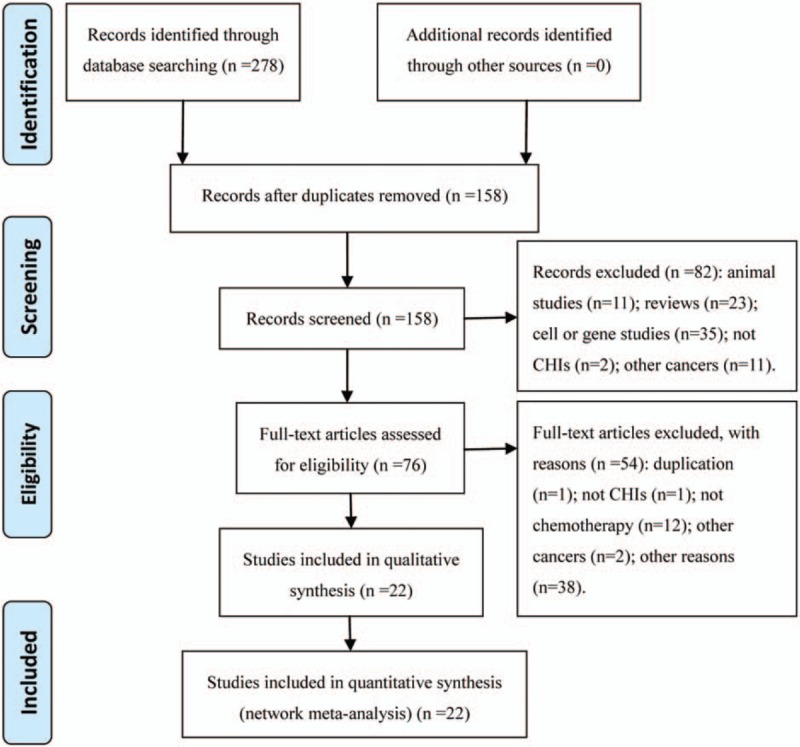
Flow chart of the search for eligible studies.

A total of 278 articles were retrieved via a primary search of the aforementioned literature databases. After reading titles and abstracts to remove the irrelevant articles and reading the full texts to remove those that did not meet the inclusion criteria, a total of 22 RCTs that evaluated CHIs combined with the chemotherapy for the treatment of pancreatic cancer were identified. In total, 9 types of CHIs were identified, including Compound kushen, Kanglaite, Kangai, Shenqifuzheng, Huanchansu, Aidi, Javanica oil emulsion, Disodium cantharidinate and vitamin B6, Astragalus polysaccharide injections. 1 RCT was published in English, and the other trials were published in Chinese (Fig. 1).

**Figure 2:**
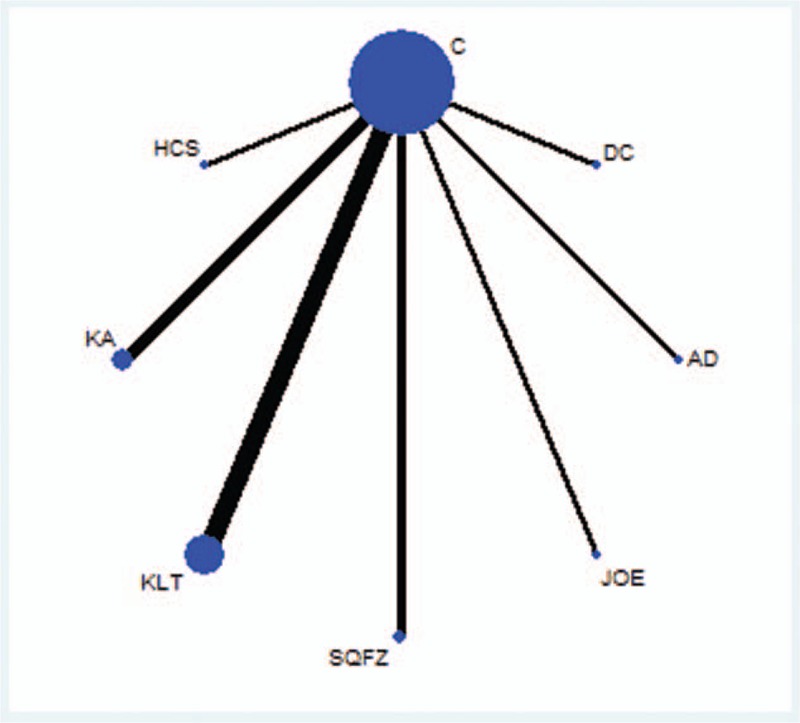
Network graph for leucopenia. Note: Node sizes indicate total sample sizes for treatments. Line thicknesses correspond to the number of trials used for comparisons. AD = Aidi, C = chemotherapy, DC = disodium cantharidinate and vitamin B6, HCS = Huanchansu, JOE = Javanica oil emulsion injection, KA = Kangai, KLT = Kanglaite, SQFZ = Shenqifuzheng.

The 22 RCTs included 9 types of CHIs and 1329 patients, among which 675 patients were in the CHIs groups and 654 were in the chemotherapy groups.^[32–53]^ All of the included RCTs reported patient numbers and ages, whereas 21 (95.45%), 10 (45.45%), 9 (40.91%) and 11 (50.00%) trials separately described the patients’ gender, tumor staging of pancreatic cancer, expected survival time and KPSs before treatment, respectively. More details regarding the individual trials are presented in Table 1. And Fig. 2 depicted a network graph of leucopenia for with 7 types of CHIs combined with chemotherapy and chemotherapy alone for pancreatic cancer.

**Table 1:**
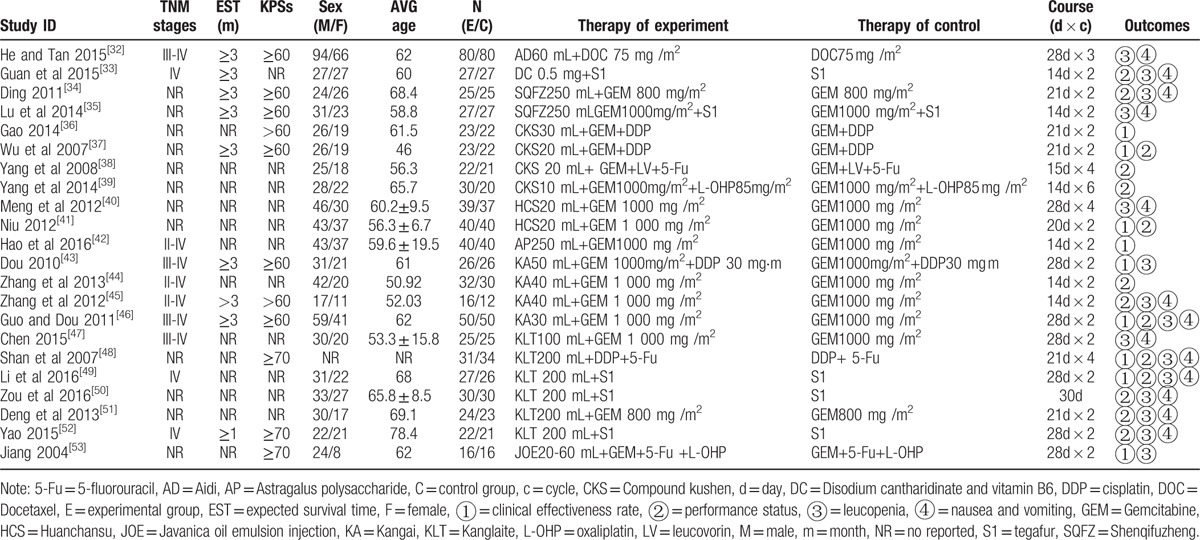
The basic characteristics of the included studies.

### 3.2 Quality assessment

**Figure 3:**
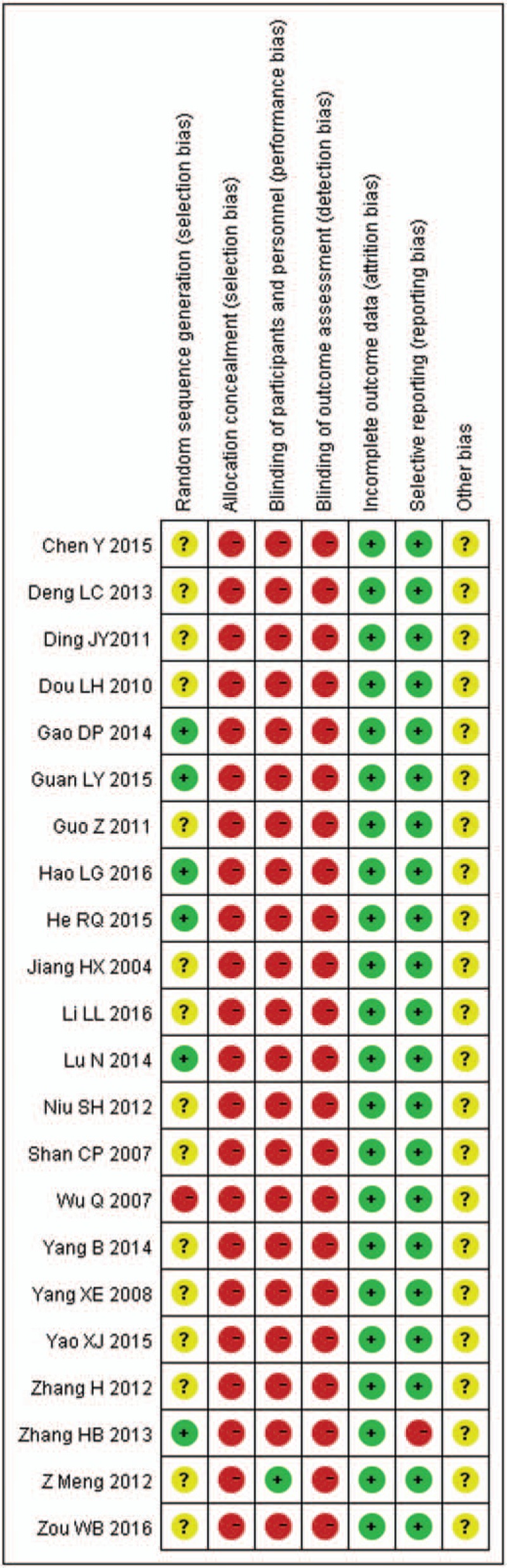
Risk of bias graph.

Although all trials mentioned randomization, only 4 RCTs (18.18%) used a random number table, 1 RCT (4.55%) used an envelope method for randomization, 1 RCT (4.55%) used the lot drawing method, and 1 RCT (4.55%) adopted the method of hospitalized time difference. Only 1 RCT (4.55%) referred to the method of blinding. Regarding allocation concealment, the included RCTs did not mention it. Except for 1 RCT had selective outcome reporting, the other 21 RCTs (95.45%) did not select outcome reporting or have incomplete outcome data. And the included RCTs did not provide information about other bias (Fig. 3). In addition, the included RCTs described the inclusion and exclusion criteria but did not mention the sample size estimation and funding. Follow-up information for 4 RCTs (18.18%) was available. As for ADRs, 15 RCTs (68.18%) evaluated the ADRs which caused by chemotherapeutic drugs. And 6 RCTs (27.27%) described the details about medical ethics.

### 3.3 Outcomes

#### 3.3.1 The clinical effectiveness rate

**Figure 4:**
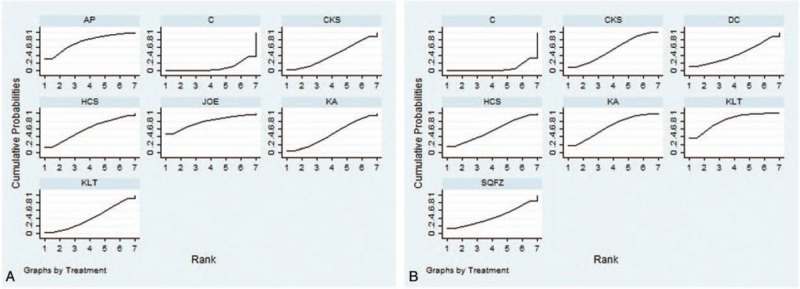
Rank of the cumulative probabilities of the clinical effectiveness rate and performance status. Note: A = the clinical effectiveness rate, AP = Astragalus polysaccharide, B = performance status, C = chemotherapy, CKS = Compound kushen, DC = disodium cantharidinate and vitamin B6, HCS = Huanchansu, JOE = Javanica oil emulsion, KA = Kangai, KLT = Kanglaite, SQFZ = Shenqifuzheng injection.

A total of 9 RCTs with 6 types CHIs reported the clinical effectiveness rate. Indirect comparisons demonstrated that the experimental groups that on the basis of chemotherapy also received Compound kushen, Huanchansu, Astragalus polysaccharide, Kangai, Kanglaite, Javanica oil emulsion injections could not experience the better clinical effectiveness rates than the control group that received the chemotherapy alone; and there were no statistically significant between-group differences. And among the CHIs groups, there was no statistically significant between-group difference. Based on the calculated probabilities for clinical effectiveness rate, the examined CHIs were ranked as follows: Javanica oil emulsion > Astragalus polysaccharide > Huachansu > Kangai > Compound kushen > Kanglaite. The results of indirect comparisons of the clinical effectiveness rate of each CHIs were shown in Table 2, and their calculated probabilities were shown in Fig. 4.

**Table 2:**
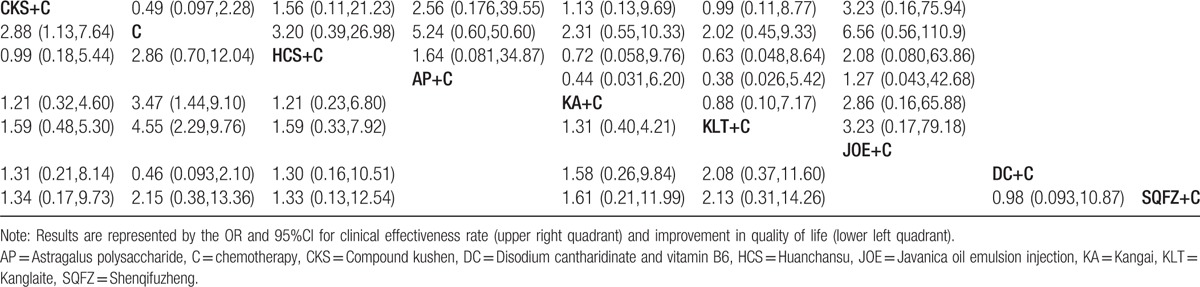
Results of the network meta-analysis of the clinical effectiveness rate (upper right quarter) and performance status (lower left quarter).

#### 3.3.2 Performance status

A total of 14 RCTs with 6 types of CHIs reported the improvement in performance status. Indirect comparisons demonstrated that the experimental groups that received Compound kushen + chemotherapy, Kangai + chemotherapy, and Kanglaite + chemotherapy experienced superior improvement in performance status relative to that of the control group which only received the chemotherapy; these between-group differences were statistically significant, with ORs and 95% CIs of 62.88 (1.13,7.64), 3.47 (1.44,9.1), and 4.55 (2.29,9.76), respectively (Table 2). However, among the CHIs groups, there were no statistically significant between-group differences. Based on the calculated probabilities for improvement in performance status, the CHIs were ranked as follows: Kanglaite > Kangai > Compound kushen > Huachansu > Disodium cantharidinate and vitamin B6 > Shenqifuzheng. The results of indirect comparisons of the improvement in performance status were shown in Table 2, and their calculated probabilities were shown in Fig. 4.

#### 3.3.3 ADRs

##### 3.3.3.1 Leukopenia

A total of 14 RCTs with 7 types of CHIs reported the leukopenia. Indirect comparisons demonstrated that the experimental groups that received Aidi + chemotherapy could relieve leukopenia than the control group which only received the chemotherapy; these between-group differences were statistically significant, with ORs and 95% CIs of 5.34 (1.7,17.05) (Table 3). And among the CHIs groups, there was no statistically significant between-group difference. Based on the calculated probabilities for leukopenia, the CHIs were ranked as follows: Aidi > Javanica oil emulsion > Disodium cantharidinate and vitamin B6 > Kangai > Shenqifuzheng > Kanglaite > Huachansu. The results of indirect comparisons of leukopenia were shown in Table 3.

**Table 3:**
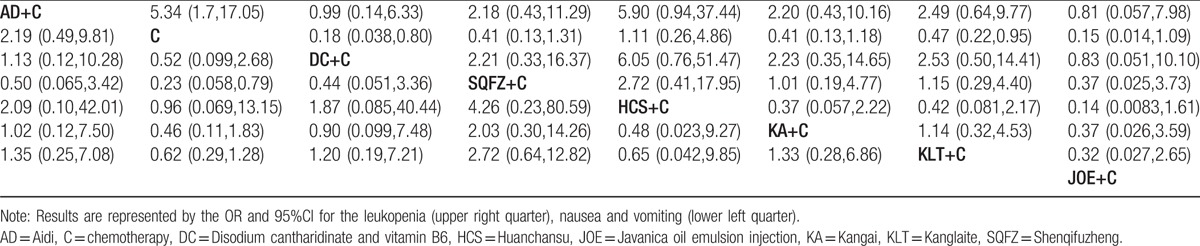
Results of the network meta-analysis of the leukopenia (upper right quarter), nausea, and vomiting (lower left quarter).

##### 3.3.3.2 Nausea and vomiting

13 RCTs with 6 types of CHIs reported the nausea and vomiting. Indirect comparisons indicated that the experimental groups that on the basis of chemotherapy also received Aidi, Disodium cantharidinate and vitamin B6, Shenqifuzheng, Huachansu, Kanglaite, and Kangai could not relieve the nausea and vomiting than the control group that received the chemotherapy alone; these between-group differences were not statistically significant (Table 3). And among the CHIs groups, there was no statistically significant between-group difference. Based on the calculated probabilities for nausea and vomiting, the CHIs were ranked as follows: Shenqifuzheng > Aidi > Kangai > Disodium cantharidinate and vitamin B6 > Kanglaite > Huachansu. The results of indirect comparisons of nausea and vomiting were shown in Table 3.

#### 3.3.4 Publication bias

**Figure 5:**
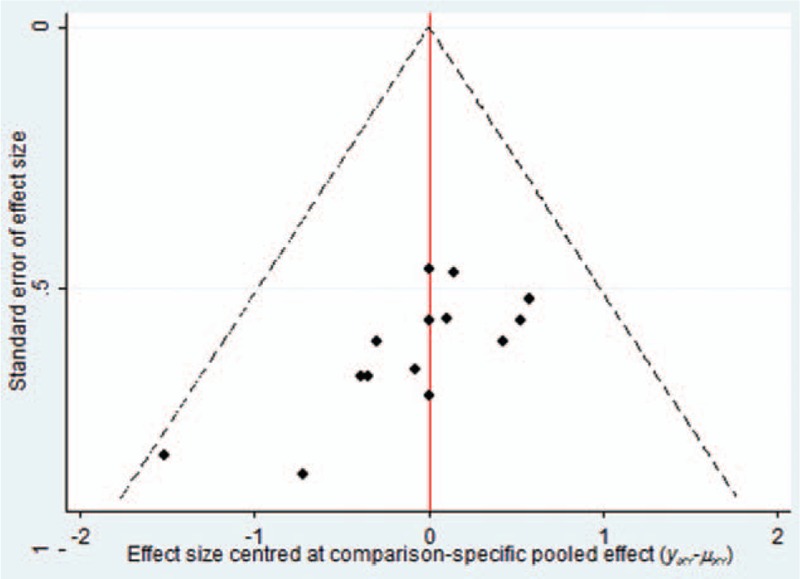
Funnel plot of performance status.

A funnel plot was used to measure the publication bias. The funnel plot of the improvement in performance status analysis showed potential publication bias of the included RCTs (Fig. 5).

#### 3.3.5 Cluster analysis

**Figure 6:**
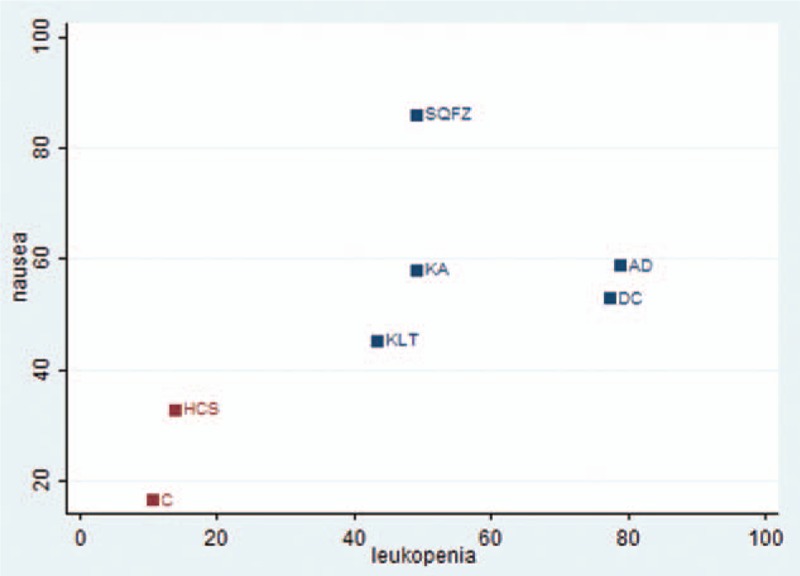
Cluster analysis plot of ADRs. Note: AD = Aidi, C = chemotherapy, DC = disodium cantharidinate and vitamin B6, HCS = Huanchansu, KA = Kangai, KLT = Kanglaite injection, SQFZ = Shenqifuzheng, X axis = leukopenia, Y-axis = nausea and vomiting.

A cluster analysis was conducted for 6 types of CHIs that reported both nausea and vomiting, and leukopenia. The plot was based on cluster analysis of SUCRA values, and each plot shows SUCRA values for ADRs. Each color represents a group of treatments that belong to the same cluster. Treatments located in the upper right corner were superior to other CHIs for relieve ADRs. The results of the cluster analysis demonstrated that the chemotherapy alone and Huachansu injection combined with the chemotherapy were inferior to relieve ADRs than the other CHIs plus chemotherapy for patients with pancreatic cancer. Aidi, Disodium cantharidinate and vitamin B6 were the most beneficial CHIs for alleviating leukopenia in combination with the chemotherapy. Shenqifuzheng was associated with having a good effect of relieving nausea and vomiting (Fig. 6).

## 4 Discussion

Our study evaluated the clinical effect, improvement in performance status, and ADRs after the application of CHIs combined with the chemotherapy for the treatment of pancreatic cancer by conducting a network meta-analysis. After analysis of the included RCTs, our study indicated that compared with the chemotherapy alone, Compound Kushen, Kangai or Kanglaite injection plus chemotherapy yielded significantly higher probability of improving the performance status. Aidi injection combined with chemotherapy was more effective in relieve leucopenia than using chemotherapy single. And these between-group differences were statistically significant. However, CHIs combined with chemotherapy could not achieve a better effect in the total clinical effect, nausea and vomiting. The cluster analysis for ADRs indicated that the chemotherapy alone and Huachansu injection combined with the chemotherapy were inferior to relieve ADRs than the other CHIs plus chemotherapy for patients with pancreatic cancer.

As one kind of malignant tumors in the digestive tract, pancreatic cancer has the features of occult clinical manifestation, rapid development, and poor prognosis. And at present, chemotherapy is still one of the effective treatments for pancreatic cancer.^[54]^ GEM has been in the first-line treatment for pancreatic cancer over 10 years,^[55]^ recently, the chemotherapy regime of GEM combined with other chemotherapeutic drugs such as 5-FU, L-OHP has been applied in the clinical studies.^[56,57]^ However, some studies found that there are 2 major obstacles in the chemotherapy treatment. One is that the chemotherapeutic drugs could lose their potency over time due to the development of multidrug resistance. The other is that chemotherapy would produce serious side effects during medical practice, and the side effects might cause systemic multi-organ damages including hematological system, circulatory system, and even nervous system.^[54,58]^ TCM may provide solutions to the above problems because of its own unique advantages: TCM has characterized by overall regulation, syndrome differentiation treatment, specimen and centralizer. And to achieve therapeutic effects to cancer, it can balance of yin and yang, promoting the body resistance and eliminate pathogenic factors by multiaspect, multilink, multitarget.^[59]^ The antitumor effectiveness of TCM injections, such as Javanica oil emulsion, Huachansu, and Aidi mainly involved shrinking the tumor along with amelioration of symptoms, thereby improving the performance status.^[60,61]^ Relevant studies has proved that Kanglaite injection can inhibits proliferation and induces apoptotic the xenografts of human pancreatic cancer, the pharmacological mechanism of its main components, coixan were anti-angiogenesis of tumors, promoting the apoptosis of cancer cells, and decreasing the enzyme activity.^[62]^ Aidi injection was extracted from *ginseng, Astragalus, Radix Acanthopanacis Semticosi, Chinese blister beetle*; it can be used to treat cancer due to its heat-clearing and detoxifying effects.^[63,64]^ Correlative studies have reported that Kangai injection was made from *ginseng, Astragalus, Sophora flavescens*, and its functions were replenishing qi and strengthening the body resistance owing to its active components, namely Astragalussaponins, ginsenoside, and matrine.^[65,66]^ Astragalus polysaccharide and ginsenoside Rg3 can inhibit tumor cell proliferation, regulate immune function and induce tumor cell apoptosis,^[67–69]^ and matrine can improve the treatment effects of advanced primary tumors and reduce the ADRs of interventional chemotherapy.^[70,71]^ Compound Kushen injection was composed of *Rhizoma Heterosmilacis Japonicae* and *Sophora flavescens*, and it had the effects of heat-clearing and damp-inhibiting, blood-cooling and toxin-relieving, stagnation-eliminating and pain-relieving.^[72]^ Moreover, validated modern pharmacological findings have shown that the anti-cancer components, matrine and oxymatrine can effectively reduce, stabilize, or even inhibit the growth of tumor, and improve the clinical symptoms of pain, fever, and fatigue.^[73–74]^

The advantages of this study were shown in the following aspects: first, this is the first network meta-analysis to compare the effectiveness and safety of CHIs in patients with pancreatic cancer. Literature searches were conducted about 22 types of CHIs which have been used for cancer treatment at the present, and the inclusion and exclusion criteria were established strictly. Second, the retrieval of this study was relatively comprehensive. On the one hand, apart from searching the database of domestic and foreign, we also search RCTs at related academic organization websites. On the other hand, the search words were divided into 3 parts: pancreatic cancer, CHIs and RCTs, the search strategy used a combination of subject words and random words. Third, the common interventions were chemotherapy of included RCTs, the criterion of the rapeutical effect met the WHO for solid tumors. Finally, this study not only analyzed the clinical effectiveness rate and the improvement of performance status, but also focused on the ADRs.

## 5 Limitation

Our study had several limitations. First, survival time was an important outcome for evaluating the curative effect of cancer, whereas in the included RCTs, only 4 trails reported the information of survival time or follow-up. Second, this study was limited by the quantity and quality of the included RCTs, the majority of RCTs included in the analysis exhibited a high risk of bias, largely due to inadequate allocation concealment and blinding. No direct head-to-head comparison was conducted between different CHIs. And the quantity of RCTs regarding CHIs for pancreatic cancer was not large. Third, all of the included RCTs were performed in patients of Asian descent; therefore, it is unclear whether the conclusions of our study apply to other populations. Despite the above limitations, our network meta-analysis provides a complete evaluation of the clinical effect, performance status, and ADRs of different CHIs for pancreatic cancer patients. However, large-sample and multicenter, head-to-head RCTs are needed to confirm these conclusions.

## 6 Conclusion

The current evidence shows that using CHIs on the basis of the chemotherapy could be beneficial for patients with pancreatic cancer in improving performance status and reducing the ADRs.

**Addditional Information**: S1 Text. Search Strategy.(DOC)

## Supplementary Material

Supplemental Digital Content
