# Supplementary material for: Network meta-analysis of Chinese herbal injections combined with the chemotherapy for the treatment of pancreatic cancer
Source: Medicine (Baltimore). 2017 May 26;96(21):e7005. doi: 10.1097/MD.0000000000007005 (PMC5457895; doi:10.1097/MD.0000000000007005)
Supplement: Supplemental Digital Content [file medi-96-e7005-s001.doc]

**S1.** S**earch strategy**

**1. Search strategy of TCM injections**

| Names of Chinese Herbal injections | English Searching Words | Chinese Searching Words |
| --- | --- | --- |
| 艾迪注射液 | Aidi | 艾迪注射液OR艾迪注射剂OR爱迪注射液OR爱迪注射剂OR注射用艾迪OR艾迪液 |
| 蟾酥注射液 | Chansu OR Toad venom | 佳素OR史君轻OR蟾毒康OR蟾酥注射液OR蟾酥注射剂 |
| 复方苦参注射液 | Compound matrine OR Compound Kushen OR Fufangkushen | 岩舒注射液OR岩舒 OR 复方苦参注射液OR 复方苦参注射剂OR 复方苦参 |
| 华蟾素注射液 | Huachansu OR Cinobufacini | 华蟾素注射液 OR 华蟾素注射剂OR华蟾素 |
| 康莱特注射液 | Kanglaite | ZCE-3静脉乳OR薏苡仁提取液OR注射薏苡仁油OR薏苡仁酯OR康莱特注射液OR康莱特注射剂 |
| 人参多糖注射液 | Renshenduotang OR Ginseng polysacchride | 安尔欣OR奥康莱OR癌得安OR长春博奥OR百扶欣OR人参多糖注射液OR人参多糖注射剂OR注射用人参多糖 |
| 痛可宁注射液 | Tongkening | 痛可宁注射液OR痛可宁注射剂OR 痛可宁 |
| 乌头注射液 | Wutou | 泰癌注射液OR乌头注射液OR乌头注射剂 |
| 消癌平注射液 | Xiaoaiping OR Marsdenia Tenacissima | 通关藤提取物OR通关藤注射液OR消癌平注射液OR消癌平注射剂OR消癌平 |
| 鸦胆子油乳注射液 | Yadanziyouru OR Javanica oil emulsion | 安体康注射液OR鸦胆子油乳注射液OR鸦胆子油乳注射剂OR鸦胆子油乳 |
| 猪苓多糖注射液 | Zhulingduotang OR Polyporus | 猪苓多糖注射液OR猪苓多糖注射剂OR注射用猪苓多糖 |
| 得力生注射液 | Delisheng | 得力生注射液OR得力生注射剂OR得力生 |
| 参芪扶正注射液 | Shenqifuzheng | 参芪扶正注射液OR参芪扶正注射剂OR参芪扶正 |
| 元秦止痛注射液 | Yuanqinzhitong | 元秦止痛注射液OR元秦止痛注射剂OR元秦止痛 |
| 黄芪多糖注射液 | Huangqi duotang OR Astragalus polysaccharides | 注射用黄芪多糖OR黄芪多糖注射液OR黄芪多糖注射剂 |
| 康艾注射液 | Kangai | 康艾液OR康艾注射液OR康艾注射剂 |
| 参附注射液 | Shenfu | 参附注射液 OR 参附注射剂 OR 注射用参附 |
| 黄芪注射液 | Huangqi OR Astragalus | 黄芪注射液OR黄芪注射剂 OR 注射用黄芪冻干粉 |
| 香菇多糖注射液 | Xiangguduotang OR Lentinan | 力提能OR 天地欣 OR香菇多糖注射液OR香菇多糖注射剂OR注射用香菇多糖 |
| 参麦注射液 | Shenmai | 参麦注射液OR参麦注射剂OR注射用参麦 |
| 榄香烯注射液 | Lanxiangxi OR Elemene | 榄香烯注射液 OR 榄香注射液 OR β-榄香烯注射液 OR 榄香烯脂质体注射液 OR 榄香烯乳注射液 |
| 斑蝥酸钠维生素B6注射液 | Sodium Cantharidinate OR Banmaosuanna | 斑蝥酸钠维生素B6注射液 OR斑蝥酸钠维生素B6注射剂 OR 艾易舒注射液 OR 艾易舒 |

**2. Search strategy of** **Pubmed**

#1 Pancreatic Neoplasms[MeSH Terms]

#2 Insulinoma[Title/Abstract]

#3 Gastrinoma[Title/Abstract]

#4 Glucagonoma[Title/Abstract]

#5 Somatostatinoma[Title/Abstract]

#6 Vipoma[Title/Abstract]

#7 Pancreatic Neoplasm[Title/Abstract]

#8 Pancreas Neoplasm*[Title/Abstract]

#9 Pancreas Cancer*[Title/Abstract]

#10 Pancreatic Cancer*[Title/Abstract]

#11 #1 OR #2 OR #3 OR #4 OR #5 OR #6 OR #7 OR #8 OR #9 OR #20

#12 Aidi[Title/Abstract]

#13 Chansu[Title/Abstract]

#14 Toad venom[Title/Abstract]

#15 Compound matrine[Title/Abstract]

#16 Fufangkushen[Title/Abstract]

#17 Compound Kushen[Title/Abstract]

#18 Huachansu[Title/Abstract]

#19 Cinobufacini[Title/Abstract]

#20 Kanglaite[Title/Abstract]

#21 Renshenduotang[Title/Abstract]

#22Ginseng polysacchride[Title/Abstract]

#23Tongkening[Title/Abstract]

#24 Wutou[Title/Abstract]

#25 Xiaoaiping[Title/Abstract]

#26 Marsdenia Tenacissima[Title/Abstract]

#27 Yadanziyouru[Title/Abstract]

#28 Javanica oil emulsion[Title/Abstract]

#29 Zhulingduotang[Title/Abstract]

#30 Polyporus[Title/Abstract]

#31 Delisheng[Title/Abstract]

#32 Shenqifuzheng[Title/Abstract]

#33 Yuanqinzhitong[Title/Abstract]

#34 Huangqi duotang[Title/Abstract]

#35 Astragalus polysaccharides[Title/Abstract]

#36 Kangai[Title/Abstract]

#37 Shenfu[Title/Abstract]

#38 Huangqi[Title/Abstract]

#39 Astragalus[Title/Abstract]

#40 Xiangguduotang[Title/Abstract]

#41 Lentinan[Title/Abstract]

#42 Shenmai[Title/Abstract]

#43 Lanxiangxi[Title/Abstract]

#44 Elemene[Title/Abstract]

#45 #12 OR #13 OR #14 OR #15 OR #16 OR #17 OR #18 OR #19 OR #20 OR #21 OR #22 OR #23 OR #24 OR #25 OR #26 OR #27 OR #28 OR #29 OR #30 OR #31 OR #32 OR #33 OR #34 OR #35 OR #36 OR #37 OR #38 OR #39 OR #40 OR #41 OR #42 OR #43 #44

#46 randomized controlled trial[Publication Type]

#47 controlled clinical trial[Publication Type]

#48 randomized[Title/Abstract]

# 49 placebo[Title/Abstract]

#50 randomly[Title/Abstract]

#51 trial[Title/Abstract]

#52 groups[Title/Abstract]

#53 "drug therapy" [Subheading]

#54 #44 OR #45 OR #46 OR #47 OR #48 OR #49 OR #50 OR #51

#55 animals[MeSH Terms]

#56 humans[MeSH Terms]

#57 #55 NOT #56

#58 #54 NOT #57

#59 #11 AND #45 AND #58

**3. Search strategy of** **Embase**

#1 random*

#2 placebo*

#3 doubl* adj blind*

#4 singl* adj blind*

#5 assign*

#6 allocat*

#7 “double-blind procedure”/exp

#8 ”randomized controlled trial”/exp

#9”single-blind procedure”/exp

#10 #1 or #2 or #3 or #4 or #5 or #6 or #7 or #8 or #9

#11 ''pancreas tumor'/exp

#12 Pancreatic Neoplasms

#13 Insulinoma

#14 Gastrinoma

#15 Glucagonoma

#16 Somatostatinoma

#17 Vipoma

#18 Pancreatic Neoplasm

#19 Pancreas Neoplasm*

#20 Pancreas Cancer*

#21 Pancreatic Cancer*

#22 #11 OR #12 OR #13 OR #14 OR #15 OR #16 OR #17 OR #18 OR #19 OR #20 OR #21

#23 aidi

#24 chansu

#25 toad AND venom

#26 compound AND matrine

#27 fufangkushen

#28 huachansu

#29 cinobufacini

#30 kanglaite

#31 renshenduotang

#32 ginseng AND polysacchride

#33 tongkening

#34 wutou

#35 xiaoaiping

#36 marsdenia AND tenacissima

#37 yadanziyouru

#38 javanica AND oil AND emulsion

#39 zhulingduotang

#40 polyporus

#41 delisheng

#42 shenqifuzheng

#43 yuanqinzhitong

#44 huangqi AND duotang

#45 astragalus AND polysaccharides

#46 kangai

#47 shenfu

#48 huangqi

#49 astragalus

#50 xiangguduotang

#51 lentinan

#52 shenmai

#53 lanxiangxi

#54 elemene

#55 #23 OR #24 OR #25 OR #26 OR #27 OR #28 OR #29 OR #30 OR #31 OR #32 OR #33 OR #34 OR #35 OR #36 OR #37 OR #38 OR #39 OR #40 OR #41 OR #42 OR #43 OR #44 OR #45 OR #46 OR #47 OR #48 OR #49 OR #50 OR #51 OR #52 OR #53 OR #54

#56 #10 AND #19 AND #52
